# Association between APOA5 polymorphisms and susceptibility to metabolic syndrome: a systematic review and meta-analysis

**DOI:** 10.1186/s12864-024-10493-x

**Published:** 2024-06-12

**Authors:** Sima Mozafari, Marziyeh Ashoori, Seyed Mahdi Emami Meybodi, Roya Solhi, Seyed Reza Mirjalili, Ali Dehghani Firoozabadi, Sepideh Soltani

**Affiliations:** 1 Yazd Cardiovascular Research Center, Non-Communicable Diseases Research Institute, Shahid Sadoughi University of Medical Sciences, Afshar Hospital, Jomhouri Blvd., Yazd, 8917945556 Iran; 2 Rasool Akram Medical Complex, Clinical Research Development Center, Tehran, Iran; 3 Department of Regenerative Medicine, Cell Science Research Center, Royan Institute for Stem Cell Biology and Technology, Academic Center for Education, Culture and Research (ACECR), Tehran, Iran

**Keywords:** Apolipoprotein A5, Metabolic syndrome, Polymorphisms, Single nucleotide polymorphism, Meta-analysis

## Abstract

**Background:**

The association between Apolipoprotein A5 (APOA5) genetic polymorphisms and susceptibility to metabolic syndrome (MetS) has been established by many studies, but there have been conflicting results from the literature. We performed a meta-analysis of observational studies to evaluate the association between APOA5 gene polymorphisms and the prevalence of MetS.

**Methods:**

PubMed, Web of Science, Embase, and Scopus were searched up to April 2024. The random effects model was used to estimate the odds ratios (ORs) and 95% confidence intervals (CI) of the association between APOA5 gene polymorphisms and the prevalence of MetS development. The potential sources of heterogeneity were evaluated by subgroup analyses and sensitivity analyses.

**Results:**

A total of 30 studies with 54,986 subjects (25,341 MetS cases and 29,645 healthy controls) were included. The presence of rs662799 and rs651821 polymorphisms is associated with an approximately 1.5-fold higher likelihood of MetS prevalence (OR = 1.42, 95% CI: 1.32, 1.53, *p* < 0.001; I^2^ = 67.1%; P-heterogeneity < 0.001; and OR = 1.50, 95% CI: 1.36–1.65, *p* < 0.001), respectively. MetS is also more prevalent in individuals with the genetic variants rs3135506 and rs2075291. There was no evidence of a connection with rs126317.

**Conclusion:**

The present findings suggest that polymorphisms located in the promoter and coding regions of the APOA5 gene are associated with an increased prevalence of MetS in the adult population. Identifying individuals with these genetic variations could lead to early disease detection and the implementation of preventive strategies to reduce the risk of MetS and its related health issues. However, because the sample size was small and there was evidence of significant heterogeneity for some APOA5 gene polymorphisms, these results need to be confirmed by more large-scale and well-designed studies.

**Supplementary Information:**

The online version contains supplementary material available at 10.1186/s12864-024-10493-x.

## Background

Metabolic syndrome (MetS) is a group of risk factors for cardiovascular disease, which include dyslipidemia, hypertension, hyperglycemia, and obesity, particularly central adiposity [1]. According to existing estimates, MetS is prevalent in approximately 31% of the world’s population [2]. MetS is associated with a 4-fold increase in coronary artery disease events [3] and a 1.2-fold increase in overall mortality [4]. Additionally, the prevalence of some cancers [5–8] higher in people with MetS compared to the general population. Given the high prevalence of MetS and its health effects, it is critical to understand the factors that influence its development.

In addition to the influence of environmental factors such as diet and exercise, inherited genetic variation is also a significant component in MetS development [9]. Single nucleotide polymorphisms (SNPs), the most common type of genetic variation, are likely to increase the risk of MetS.

The position of SNPs can determine how they affect the structure and quantity of gene products, which could explain the difference in susceptibility to various diseases [10]. Some SNPs located in a coding area or promoter region could explain the difference in susceptibility to various diseases [11]. Genome-wide association studies (GWAS) have identified several candidate genes, including apolipoprotein A5 (APOA5), which may influence susceptibility to metabolic syndrome (MetS) and its components [12–14].

The APOA5 locus is on chromosome 11q23 and has 4 exons that code for APOA5. It is next to the APOA1/APOC3/APOA4 gene cluster. APOA5, a 366 amino acid protein identified in triglyceride (TG)-rich lipoproteins and high density lipoprotein (HDL) particles [15–18], is a potent regulator of plasma TG and HDL-C levels [19].

Several SNPs in APOA5 have been reported to have significant effects on MetS components. The most commonly reported polymorphisms to be associated with TG levels, plasma HDL levels, and other MetS traits are − 1131T > C, c56C > G, and c553G > T [19–22].

So far, three meta-analyses have been published on the relationship between APOA5 polymorphisms and MetS [23–25]. In the past decade, there were two meta-analysis published [23, 24], but then 16 new studies related to the topic were published [15, 19, 21, 22, 25–36]. The other meta-analysis [25] has only focused on the North African population. Furthermore, in these meta-analyses, only two common polymorphisms, including − 1131T > C and c56C > G, have been examined [23–25]. While recent studies evaluated the association of emerging polymorphisms, such as c553G > T, -3 A > G, -12238T > C, and c1259T > C with MetS [19, 21, 22, 26, 35]. Hence, the results of these studies are not conclusive and should be interpreted with caution. Considering the limitations of previous meta-analyses and the importance of scientific updates in this field, the present study was conducted to investigate the relationship between different APOA5 variants and MetS in the adult population through a systematic review and meta-analysis of observational studies.

## Methods

Our systematic review and meta-analysis were performed according to the Preferred Reporting Items for Systematic Reviews and Meta-Analyses (PRISMA) [37]. We have registered the protocol of this systematic review on the PROSPERO website (registration number: CRD42023461249).

### Search strategy

The electronic databases PubMed, Web of Science, Embase, and Scopus were searched before April 15, 2024, to find studies that looked into the link between APOA5 gene polymorphisms and MetS risk in adults. Also, we have not considered any restrictions on the language or publication date. The references from the selected eligible articles and previous meta-analyses were screened for other relevant articles. The complete strategy, from searching in the databases to selecting eligible studies, is shown in Supplementary Table S1.

### Inclusion and exclusion criteria

Studies were included if they met all of the following criteria: (1) all published observational studies (cross-sectional, cohort, case-control, and baseline of controlled clinical trials) that were done on adults; (2) showed a link between at least one APOA5 polymorphism and the prevalence of metabolic syndrome; (3) gave either the odds ratio (OR) and 95% confidence intervals (CI) or enough information to calculate OR and CI; and (4) used a validated definition to make the MetS diagnosis.

If studies were conducted on the same population, the study with a larger sample size was selected. Studies were excluded if they were performed among pregnant or lactating women, or if they lacked sufficient data to determine allele or genotype frequencies for APOA5. Two authors (SM and SMEM) screened the title, abstract, and full text of all retrieved references based on inclusion and exclusion criteria.

### Data extraction

The following data were extracted by two authors (SM, SMEM) and then cross-checked by a second author (SS): the first author, year of publication, country, age, gender, number of total participants and number of participants with MetS, the definition of MetS, genotyping method, the presence of HWE and APOA5 variants, and confounding factors.

### Risk of bias assessment

The quality of each study was assessed using the Newcastle-Ottawa (NOS) scale with a maximum score of 9 [38]. According to the scale, studies were classified into three levels: high quality (7–9 points), medium (4–6 points), and low quality (1–3 points).

### Statistical analysis

The ORs and their 95% CIs of the prevalence of metabolic syndrome among polymorphisms of the apolipoprotein APOA5 gene were extracted from each study. The natural logarithm scale of OR and its CI as the effect size were included in the meta-analysis under two possible genetic models: the dominant model and the recessive model. For studies that did not provide sufficient data for meta-analysis, we calculated the ORs and 95% CIs as the odds of having metabolic syndrome in the variant allele-positive genotypes versus wild-type homozygous genotype (aa + Aa vs. AA) in the dominant model and metabolic syndrome in the variant homozygous genotype vs. the rest (aa vs. AA + Aa) in the recessive model with approaches that suggested in previous studies [39, 40]. The effect size was pooled by the DerSimonian and Laird method [41] using the random effects model, which assumes within-study sampling errors and between-study variances. Statistical heterogeneity between studies was evaluated using Cochran’s Q test and I-squared (I^2^) [42]. The heterogeneity was regarded as low, moderate, and high when the I2 values were 25%, 50%, and exceeded 75%, respectively [43]. To explore the potential sources of heterogeneity, subgroup analyses were conducted, stratified by geographic location, sex, study quality, sample size, MetS definition, and adjustment for potential confounding factors (including age, BMI, and smoking). Subgroup analysis was performed only for the dominant models. The deviation from HWE for each study was assessed for the control group using the chi-squared test. The sensitivity analyses were conducted by excluding studies from the meta-analysis one by one to test the robustness of each association. Possible publication bias was assessed using Egger’s test [44] and Begg’s test [45], with the results considered to indicate publication bias at *P* < 0.10. We also visually checked funnel plots for asymmetry. All statistical analyses were performed using STATA, version 17 (Stata Corp., College Station, TX). P values less than 0.05 were considered statistically significant.

## Results

### Literature search

Our initial search yielded 730 articles, of which 242 were duplicates and 416 were unrelated after screening the title or abstract. After evaluating 71 full-text articles, 39 were excluded (reasons provided in Supplementary Table S2). The update search identified one additional eligible article, resulting in a final selection of 30 studies for the meta-analysis [11, 15, 19, 21, 22, 24–36, 46–57].

**Fig. 1:**
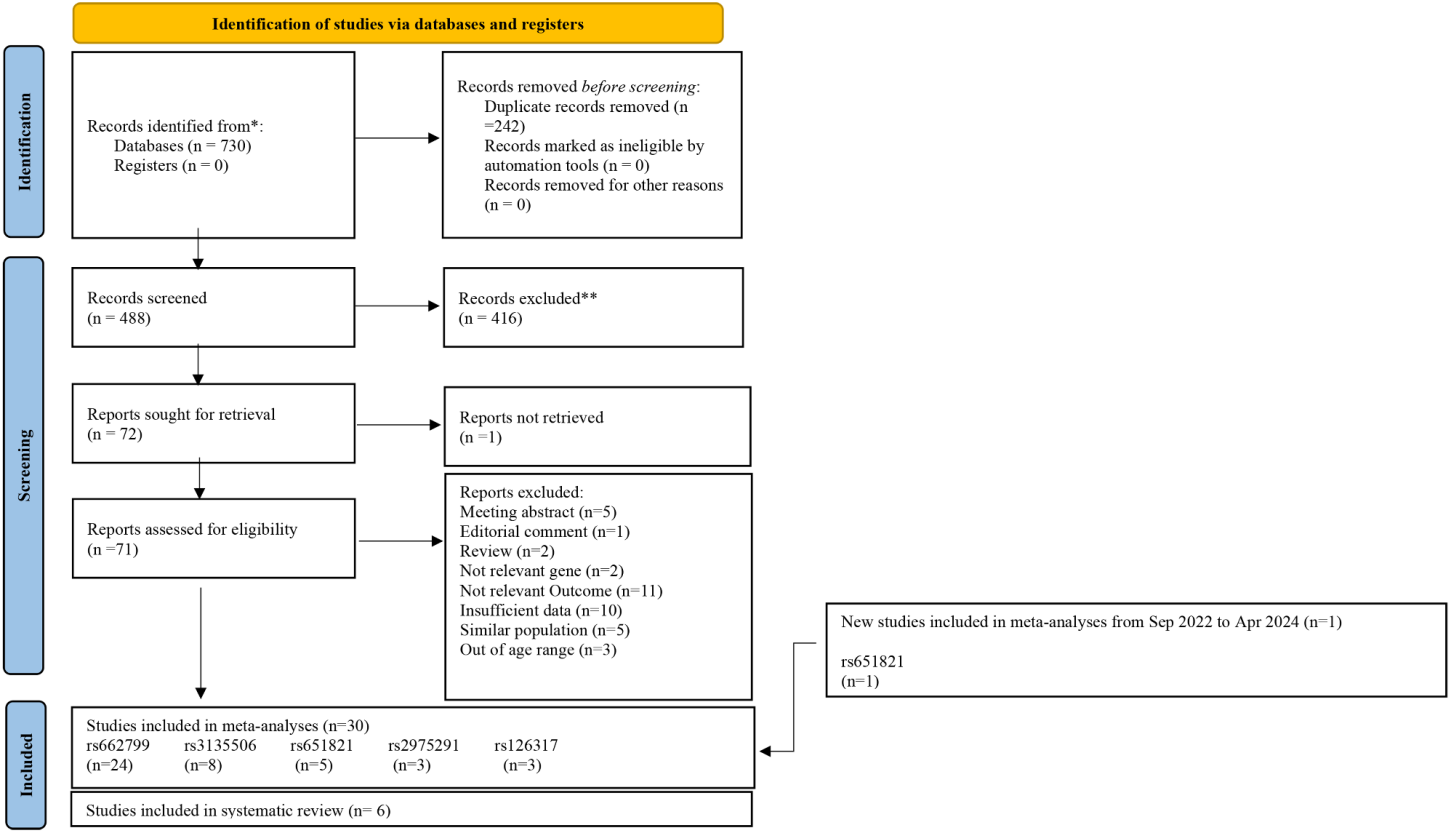
Flow diagram for the eligible study selection process

Six studies were also included for systematic review because the number of studies related to other APOA5 polymorphisms was insufficient for meta-analyses [19, 50, 58–60]. The study screening process is shown in Fig. 1.

### Studies characteristics

The general characteristics of the included studies are shown in Table 1. Our meta-analysis contained a total of 54,986 subjects, including 25,341 MetS cases and 29,645 healthy controls. All studies have cross-sectional design. Most studies were from Asia (*n* = 17) [11, 15, 22, 24, 26, 29–32, 34–36, 48, 49, 52, 55, 57] and Europe (*n* = 8) [27, 33, 46, 47, 50, 51, 54, 56], while we found just one study that was conducted in the USA [53], and four studies that were from Africa [19, 21, 25, 28]. The age range of the participants was 18 to 79, and the majority of studies were performed among both sexes, while three studies included only women [31, 32, 51]. Twenty-two studies were consistent with Hardy-Weinberg equilibrium (HWE) [11, 21, 22, 24, 25, 27, 29, 30, 32–35, 46, 48–51, 53–57]. However, in three studies, HWE was not in equilibrium [15, 36, 47], and in six studies, HWE was not reported [19, 24, 26, 28, 31, 52]. Seventeen studies used NCEP ATP III criteria [11, 22, 27, 28, 31–34, 46, 47, 49–54, 56], ten studies used IDF criteria [15, 19, 21, 24, 25, 29, 30, 35, 36, 48], and three studies used modified definition for identifying MetS [26, 55, 57]. Several techniques were used to identify the genotypes, such as the Polymerase Chain Reaction -Amplification Refractory Mutation System (PCR-RFLP) [15, 19, 22, 28, 31, 46, 48–50, 54, 56], Real-Time PCR Taqman [27, 33, 34, 51, 53], PCR and sequence-specific oligonucleotide probes (PCR-SSOP) [11, 30, 57], Mass Array [29, 52, 55], Matrix-Assisted Laser Desorption/Ionization (MALDI-TOF) [35, 47], Kaspar technology [21, 25], SNP Integration Tool (SNP-IT) assay [32], and bead-based microarray tech [24, 26, 36].

**Table 1:** Characteristics and expressing the number of people with Metabolic Syndrome and control group in the included studies

Author, Year	Country	Sex/ Number	MetS definition/ (number)	Age	HWE (Yes, No and NM)	Adjustment	Measurement methods	Polymorphism
Ajjemami,2015	Morocco	Both/ 281	IDF/ 176	20–60	NM	Age, Sex, BMI	PCR-RFLP	-1131T > C c56C > G c553G > T 1259T > C
Alipour, 2023	Iran	Both/ 4546	JIS/ 2343	36.50	NM	-	Bead Based Microarray Tech	-3 A > G
Dallongeville,2008	France	Both/ 3138	ATP III/ 932	50.83	Yes	-	PCR-RFLP	c56C > G 12238T > C
de Luis, 2021	Spain	Both/ 1002	ATP III/ 468	23–62	Yes	Age, Sex	Real-Time PCR Taqman	-1131T > C
Fathy,2012	Egypt	Both/ 90	ATP III/ 60	51.33	NM	No	PCR-RFLP	-1131T > C
Fiaz,2019	Pakistan	Both/ 705	IDF/ 350	41.3	Yes	Age, Sex	Mass Array	-1131T > C
Grallert, 2007	Germany, Austria	Both/ 3004	ATP III/ 1031	40–74	No	-	MALDI-TOF-MS	-1131T > C c56C > G
He,2011	China (YAO and HAN)	Both/ 2510	IDF/ 1160	57.20	Yes	No	PCR-RFLP	12238T > C
Hechmi,2020	Tunis	Both/ 592	IDF/ 294	54.47	Yes	Age, Sex and BMI	Kaspar Technology	-1131T > C
Hiramatsu,2012	Japan, Korea	Both/ 5121	IDF/ 4019	61.80	Yes	Age, Sex	PCR-SSOP	-1131T > C
Hsu,2008	Taiwan	Both/ 615	ATP III/ 115	45.75	Yes	Age, Sex, Smoking status, Regular Exercise, WHR	PCR-RFLP	-1131T > C
Kefi,2017	Tunis	Both/ 594	IDF/ 295	54.56	Yes	Age, Sex and BMI.	Kaspar Technology	c56C > G -3 A > G
kim,2012	Korea	Female/ 307	ATP III/ 103	62.48	NM	Metabolic syndrome risk factors	PCR-RFLP	-1131T > C
Kim,2016	Korea	Both/1074	ATP III/ 415	49.08	Yes	Age, Sex	PCR-RFLP	-1131T > C 12238T > C
Kisfali,2010	Hungary	Both/ 627	ATP III/ 343	59.73	Yes	-	PCR-RFLP	-1131T > C c56C > G 1259T > C
Komurcu-Bayrak,2008	Turkey	Female/ 804	ATP III/ 396	54.10	Yes	-	ReaL-Time PCR-Taqman	-1131T > C, c56C > G
Lim, 2014	Korea	Female/ 1128	ATP III/ 164	20–59	Yes	-	SNP-IT assay	-1131T > C
Lim, 2016	Korea	Both/ 324	ATP/ 70	20–81	NM	Age, Sex	SNP Array	-3 A > G
Mattei,2009	USA	Both/ 802	ATP III/ 534	57.80	Yes	Age, Sex, Smoking status, Drinking, Population admixture, Medication use	ReaL-Time PCR-Taqman	-1131T > C c56C > G
Niculescu,2010	Romania	Both/ 279	ATP III/ 188	47.84	Yes	-	PCR-RFLP	-1131T > C c56C > G
Novotny,2014	Czech Republic	Both/ 590	ATP III/ 146	50.88	Yes	Age, Sex	ReaL-Time PCR-Taqman	-1131T > C
Ong,2011	China (Hong Kong, Guangzhou)	Both/ 3282	Harmonised definition/ 720	56.70	Yes	Age, Sex, Smoking status, Drinking, Education	Mass Array	-1131T > C
Song,2013	Korea	Both/ 2901	ATP III/ 1004	47.76	Yes	Age, Sex	ReaL-Time PCR-Taqman	-1131T > C
Vasilopoulos,2011	Greece	Both/ 90	ATP III/ 30	29.10	Yes	-	PCR-RFLP	-1131T > C
Wu,2016	China	Both/ 3850	IDF/ 1813	18–79	Yes	Age, Sex	MALDI-TOF-MS	-1131T > C -3 A > G c553G > T
Xu,2013	China	Both/ 1840	IDF/ 905	57.56	NM	Age, Sex	Bead Based Microarray Tech	-1131T > C
Yamada,2007	Japan	Both/ 2417	AHA/NHLBI/ 1522	65.61	Yes	Age, Sex, Smoking status	PCR_SSOP	-3 A > G c553G > T
Yamada,2007	Japan	Both/ 1788	ATP III/ 1017	64.06	Yes	Age, Sex, Smoking status	PCR-SSOP	-1131T > C
Yeh,2020	Taiwan	Both/ 10,285	IDF/ 4528	49.99	No	Age, Sex	Bead Based Microarray Tech	-1131T > C
Zafar,2019	Pakistan	Both/ 400	IDF/ 200	46.86	No	-	PCR-RFLP	-1131T > C

### Quality assessment

The findings of the quality evaluation revealed that 23 of the included studies were of high quality [15, 19, 21, 22, 24–27, 29, 30, 33–36, 46, 47, 49–53, 55, 57], while five were of moderate level [11, 28, 32, 48, 54]. The remaining studies were found to have low quality [31, 56] (Supplementary Table S3).

### Association between rs662799 (-1131 T > C) and MetS

**Fig. 2:**
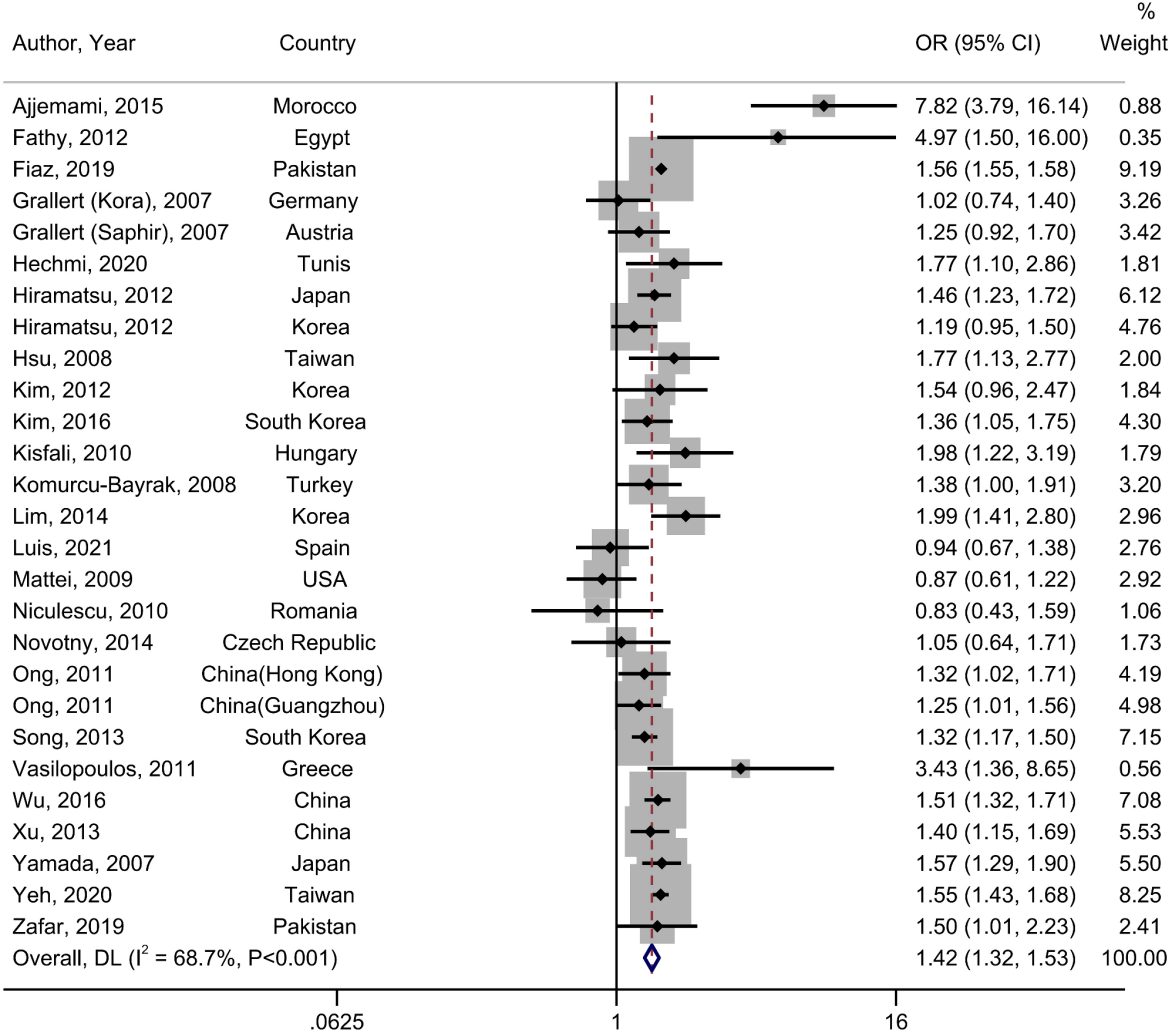
Prevalence of metabolic syndrome associated with rs662799. The black square and horizontal line represent the study-specific odds ratio (OR) and 95% CI, respectively; the area of the black square is proportional to the specific-study weight to the overall meta-analysis. The center of the open diamond presents the pooled OR and its width represents the pooled 95% CI. Weights are from random-effects analysis

The meta-analysis of the association between rs662799 polymorphism and MetS (*n* = 24 studies, 19,019 MetS and 22,438 controls) revealed a significant relationship between rs662799 polymorphism and the MetS prevalence in the dominant model (OR = 1.42, 95% CI: 1.32, 1.53; I^2^ = 68.7%; P-heterogeneity < 0.001) (Fig. 2) and recessive models (OR = 1.80, 95% CI: 1.65, 1.96; I^2^ = 0%; *P-heterogeneity* = 0.65). (Table 2) [11, 15, 19, 22, 24, 25, 27–36, 47, 49–51, 53–56]. There is a slightly more pronounced increase in studies conducted in females and studies that used IDF criteria for MetS definition. Subgroup analysis indicated that the relationship was found in Africa (OR = 3.92, 95% CI: 1.35, 11.39, *P-heterogeneity* = 0.002), Asia (OR = 1.56, 95% CI: 1.54, 1.57, *P-heterogeneity* = 0.74), and East Asia (OR = 1.44, 95% CI: 1.36, 1.53, *P-heterogeneity =* 0.22). The findings of subgroup analyses are shown in Supplementary Table S4.

**Table 2:** Summary of the overall results for APOA5 polymorphisms under the dominant and recessive models

Polymorphisms		Studies (*n*)	Participants/ Events (*n*/*n*)	Meta-analysis		Heterogeneity		
				OR (95%CI)	*P* effect	Q statistic	*P* within group	I^2^ (%)
**rs662799 (-1131 T > C)**								
Dominant (TC + CC vs. TT)		24	41,457/19,024	1.42 (1.32, 1.53)	< 0.001	83.07	< 0.001	68.7
Recessive (CC vs. TT + TC)		15	32,232/12,930	1.80 (1.65, 1.96)	< 0.001	13.26	0.65	0.0
**rs3135506 (c56 C > G)**								
Dominant (CG + GG vs. CC)		8	9509/3876	1.37 (1.21, 1.55)	< 0.001	5.18	0.74	0.0
Recessive (GG vs. CC + CG)		4	6997/2415	1.93 (0.97, 3.85)	0.06	1.16	0.88	0.0
**rs651821 (− 3 A > G)**								
Dominant (AG + GG vs. AA		5	11,731/6043	1.50 (1.36, 1.65)	< 0.001	5.37	0.25	25.5
Recessive (GG vs. AA + AG)		5	7185/3700	1.82 (1.56, 2.13)	< 0.001	2.13	0.71	0.0
**rs2075291 (c 553 G > T)**								
Dominant (GT + TT vs. GG)		3	6548/3511	1.43 (1.14, 1.81)	0.002	3.38	0.18	40.8
Recessive (TT vs. GG + GT)		3	6548/3511	1.17 (0.39, 3.51)	0.78	4.41	0.11	54.7
**rs126317 (-12,238 T > C)**								
Dominant (TC + CC vs. TT)		3	6722/2507	1.08 (0.79, 1.47)	0.64	22.83	< 0.001	86.9
Recessive (CC vs. TC + TT)		3	6722/2507	0.97 (0.70, 1.34)	0.85	19.14	< 0.001	84.3

OR, Odd Ratio; P, P-value; I^2^, I-squared

All OR values have a 95% confidence interval

### Association between rs3135506 (c56 C > G) and MetS

**Fig. 3:**
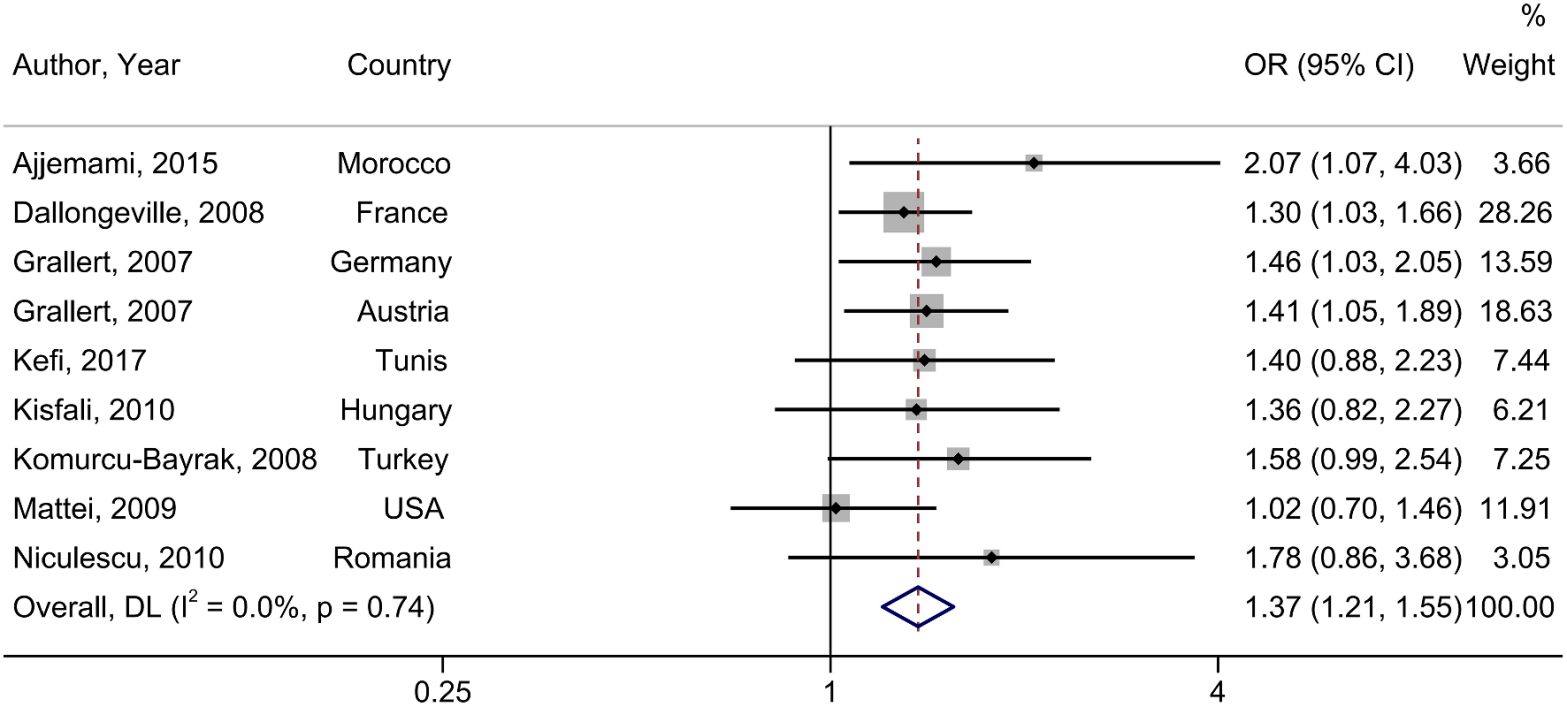
Prevalence of metabolic syndrome associated with rs3135506. The black square and horizontal line represent the study-specific odds ratio (OR) and 95% CI, respectively; the area of the black square is proportional to the specific-study weight to the overall meta-analysis. The center of the open diamond presents the pooled OR and its width represents the pooled 95% CI. Weights are from random-effects analysis

The pooled results of the 8 studies (3876 MetS and 5633 controls) showed that rs3135506 is significantly related to increased MetS prevalence in the dominant model (OR = 1.37, 95% CI: 1.21, 1.55; I^2^ = 0%, *P-heterogeneity* = 0.74) (Fig. 3) and association was not significant in the recessive model (OR = 1.93, 95% CI: 0.97, 3.85; I^2^ = 0%; *P-heterogeneity* = 0.88) (Table 2) [19, 21, 46, 47, 50, 51, 53, 54]. The results of subgroup analyses are shown in Supplementary Table S5.

### Association between rs651821 (− 3 A > G), rs2075291 (c 553 G > T), rs126317 (-12,238 T > C) and MetS

**Fig. 4:**
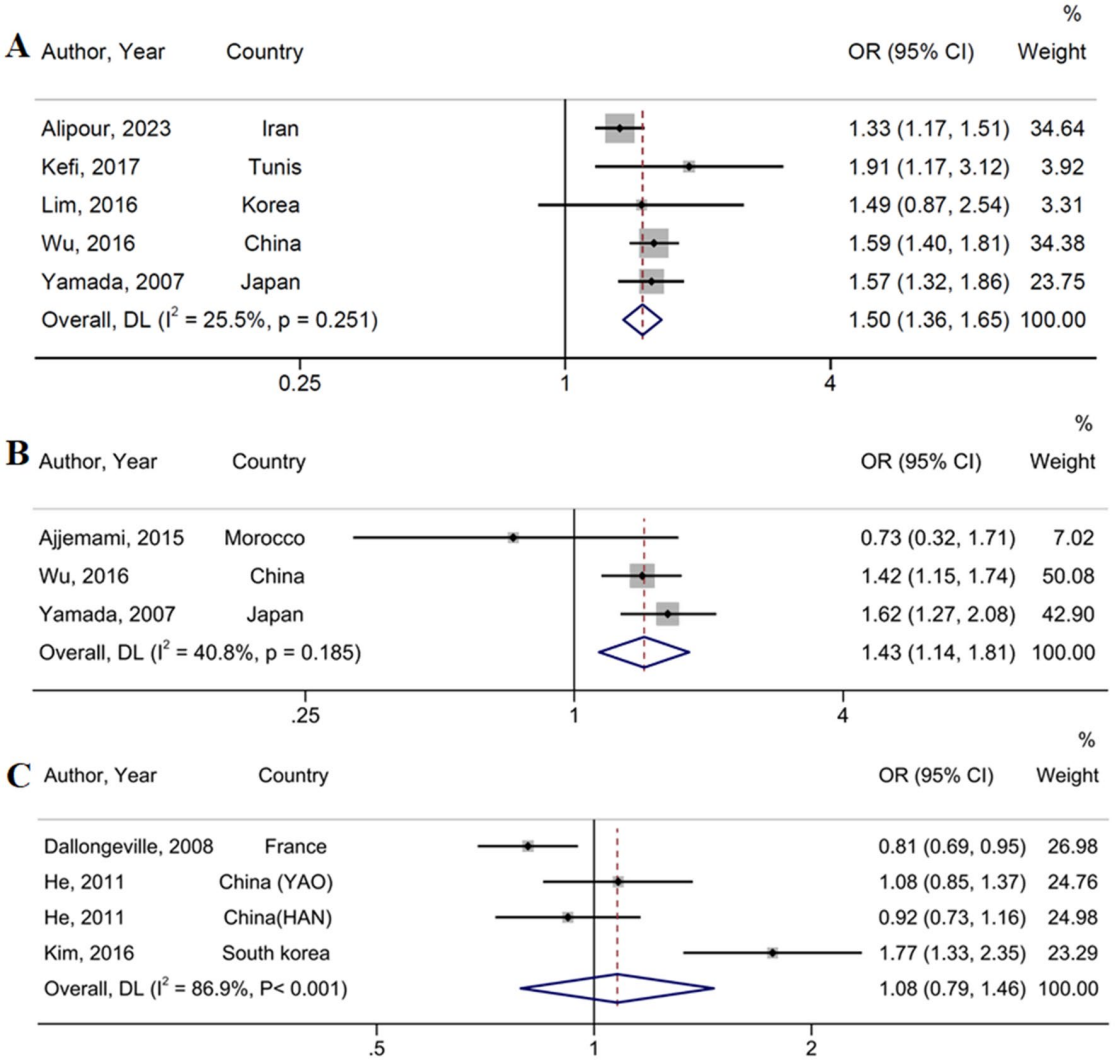
Prevalence of metabolic syndrome associated with rs651821 (**A**), rs2075291 (**B**), rs126317 (**C**). The black square and horizontal line represent the study-specific odds ratio (OR) and 95% CI, respectively; the area of the black square is proportional to the specific-study weight to the overall meta-analysis. The center of the open diamond presents the pooled OR and its width represents the pooled 95% CI. Weights are from random-effects analysis

The APOA5 polymorphism rs651821 was shown to be positively associated with MetS prevalence in both dominant (OR = 1.50, 95% CI: 1.36, 1.65; I^2^ = 25.5%, *P-heterogeneity* = 0.25) (Fig. 4 (A)) and recessive models (OR = 1.82, 95% CI: 1.56, 2.13; I^2^ = 0%, *P-heterogeneity* = 0.71) (Table 2) [21, 26, 35, 52, 57]. Additionally, the rs2075291 polymorphism was found to be associated with MetS risk in the dominant model (OR = 1.43, 95% CI: 1.14, 1.81; I^2^ = 40.8%, *P-heterogeneity* = 0.18) (Fig. 4(B)) [19, 35, 57]. However, no significant relationship between MetS risk and polymorphisms rs126317 (dominant model OR = 1.08, 95% CI: 0.79, 1.47, I^2^ = 86.9, %, *P-heterogeneity* < 0.001) (Fig. 4(C)), and recessive model OR = 0.97, 95% CI: 0.70, 1.34, I^2^ = 84.3, *P-heterogeneity* < 0.001) were observed (Table 2) [22, 46, 48]. Due to the low number of original studies included in the present meta-analysis for this polymorphism, we could not perform subgroup analysis to explore the sources of heterogeneity.

### Publication bias and sensitivity analyses

The pooled ORs were not altered by omitting any single study in the dominant model for rs662799 and rs3135506, indicating that our results were statistically robust. In addition, further analysis revealed that heterogeneity for rs662799 under dominant model remained constant even after excluding studies with a sample size of less than 100 (OR = 1.41, 95% CI: 1.31, 1.51, I² = 68.6%, *P-heterogeneity* < 0.001) [19, 28], a high sample size (OR = 1.41, 95% CI: 1.30, 1.53, I² = 69.9%, *P-heterogeneity* < 0.001) [36], obese people (OR = 1.43, 95% CI: 1.32, 1.55, I² = 68.3%, *P-heterogeneity* < 0.001) [27, 36], and and those conducted exclusively in postmenopausal women (OR = 1.40, 95% CI: 1.30, 1.51, I² = 70.4%, *P-heterogeneity* < 0.001) [31, 32].

No publication bias was observed in the association rs662799 with MetS (Egger’s test, *P* = 0.12 and Begg’s test, *P* = 0.21) and rs3135506 with MetS (Egger’s test, *P* = 0.14 and Begg’s test, *P* = 0.12) in dominant model.

### Association between other APOA5 SNPs and MetS

After reviewing the included studies, it was discovered that there are other APOA5 variants that may be involved in the MetS susceptibility. Two studies showed a strong positive association between rs2266788 and the risk of MetS [59, 60]. While Ajjemami and colleagues did not report any significant association between this variant and MetS [19]. Since the mentioned studies did not provide enough data, it was not possible to perform a meta-analysis for this SNP. Flores-Viveros et al. [58], and Kisfali et al. [50], investigations have identified rs964184 and IVS3 + G476A as genetic variants that are associated with an increased risk of MetS. However, no significant association was found between rs17120035, rs9804646, rs1729410, or rs633389 and the development of MetS [55].

## Discussion

### Principle findings

The current meta-analysis revealed substantial associations between certain APOA5 polymorphisms, including rs662799 and rs651821, in both dominant and recessive models, as well as the dominant models of rs3135506 and rs2075291. Studies that investigated the dominant model of rs662799 polymorphism showed a slightly more pronounced increase in MetS prevalence among females, as did studies that used IDF criteria for MetS definition. However, the recessive models of rs3135506 and rs2075291, as well as both the dominant and recessive models of rs126317, did not show any association with MetS.

### Comparison with previous meta-analyses

The polymorphisms rs662799 and rs3135506 have been investigated in previous meta-analyses regarding the risk of MetS [23–25]. In these meta-analyses, the mentioned SNPs have been shown to be associated with an increased risk of MetS, consistent with our findings. However, as mentioned previously, these meta-analyses had some limitations [23–25]. Firstly, these meta-analyses were published in the past decade [23, 24], and but 16 studies were published after that [15, 19, 21, 22, 25–36]. Secondly, some of these meta-analyses were specifically focused on a certain demographic, namely the African population [25]. Consequently, the generalizability of their findings is restricted. The present work is the first meta-analysis to evaluate the relationship between other APOA5 SNPs, such as rs651821, rs2075291, and rs126317, and the risk of MetS.

### Potential underlying mechanisms

The results of our study show that polymorphisms located in the promoter area (rs662799 and rs651821) and the codon area (rs3135506 and rs2075291) are more likely to be linked to MetS susceptibility. APOA5 which function as a ligand for TG-rich lipoprotein, is a key regulator of plasma triglycerides. ApoA5 is a 366 amino acid protein primarily synthesized in hepatic cells and plays a vital role in lipid metabolism [61, 62]. Even though ApoA5 has low concentrations in the blood, its loss can impair lipoprotein lipase activity and cause an increase in TG levels [61, 62]. In addition, a lack of ApoA5 can disrupt the removal of remaining lipoproteins from the bloodstream, as it plays a role in assisting LDL receptors and heparan sulfate proteoglycans [61]. As a result, APOA5 polymorphism can lead to hypertrigiceridima which is proposed as a pivotal mechanism responsible for the increased susceptibility to MetS in relation to genetic variability at the APOA5 locus.

Although ApoA5 is not expressed in adipose tissue, it may still play a role in the accumulation of triglycerides in adipocytes [62]. The accumulation of TG in adipocytes can lead to a rise in resistin, TNF-α, and interleukin 6, which can result in insulin resistance [63]. Insulin resistance is the main notion behind MetS, and it may be responsible for the majority of its components [64].

Hypertension is another component of MetS that hypertriglyceridemia can influence it through some possible mechanisms, including disturbance of the vasodilation mechanisms leading to vascular resistance, stimulating aldosterone production, and inducing insulin resistance, which is caused by stimulation of the sympathetic nervous system activity and renin–angiotensin system [65–68]. APOA5 appears to be directly or indirectly involved in most MetS components and has an association with the development of MetS.

APOA5 deficiency can exert some influences on obesity, as an additional element of metabolic syndrome. Given that APOA1, APOC3, and APOA4 are genes associated with obesity and are grouped together in the same cluster as APOA5, it is possible that mutations in one gene within the cluster may be caused by genetic variations in another gene [69]. Furthermore, it can potentially reduce satiety-related signals and contribute to obesity as a result of consuming a large number of calories [70].

The HDL is another component of metabolic syndrome that might be affected by ApoA5 loss. The ApoA5 may affect HDL levels through TG metabolism, as there is a known negative link between triglyceride and HDL-C levels [71, 72]. Moreover, ApoA5 protein is a component of HDL particles, and its absence might result in unstable HDL that is easily eliminated from serum [72]. Furthermore, Apoa5 mutation can result in impaired reverse cholesterol transport and abnormal HDL maturation, resulting in a decrease in the total amount of HDL [73].

### Implications from subgroup analyses

Our findings demonstrated that the rs662799 polymorphism is associated with metabolic syndrome (MetS) prevalence in both African and East Asian populations under the dominant model. The association observed in African populations should be interpreted with caution due to the limited number of studies (accounting for only 4% of the overall effect size). Previous meta-analysis have confirmed that APOA5 variants increase the risk of MetS in East Asian and non-white populations [23]. These discrepancies may be partially explained by factors such as differing gene-gene or gene-environment interactions [74], linkage disequilibrium patterns, and minor allele frequencies of the APOA5 SNPs [74, 75].

We also found a sex-specific association between the rs662799 polymorphism and MetS prevalence, with the association more evident in female participants. This association may be potentially justified by the fact that most female participants were in a post-menopausal status and did not benefit from exposure to female hormones, especially estrogen, which protects against visceral fat accumulation, insulin resistance, hypertension, and dyslipidemia [77].

The association between the rs662799 polymorphism and the prevalence of MetS is also influenced by the definition of MetS, with a stronger association observed when employing the IDF criteria. The IDF definition emphasizes central obesity and employed stricter cut-offs for specific MetS components, so the prevalence of MetS is typically higher when diagnosed using this definition than when using others [78].

Moreover, our results indicated that the associations of rs662799 and rs651821 with MetS were slightly stronger in the recessive models compared to the dominant models, indicating that wild-type alleles may be capable of partially attenuating the effect of mutant alleles in heterozygous individuals. Wu et al. concluded that the rs662799 and rs651821 variations are more closely linked to MetS when examined under a recessive genetic model than a dominant model [35]. It is suggested that if two copies of the allele at rs662799 or rs651821 are present, it may have a more significant effect on the risk of MetS [35]. Similarly, the results of other studies on rs662799 revealed that the concentration of ApoA5 in people with the C/C genotype is lower than that with the T/C and T/T genotypes, therefore, it is assumed that the recessive model has a higher risk of MetS than the dominant model [35, 79].

### Future perspectives

It has been shown that different genes may be associated with the MetS developing, and some genes also reinforce each other’s effects [80]. Therefore, it is crucial to consider gene-gene interactions to obtain logical and reliable outcomes. Aside from the genetic component, environmental interactions, and epigenetic factors could play significant roles in the underlying mechanism of MetS [35, 81]. Therefore, future studies on the dysregulation of epigenetic factors in MetS would provide a deeper insight into MetS pathophysiology.

### Strengths and limitations

The present meta-analysis has some strengths. This is the first comprehensive meta-analysis that investigates the majority of APOA5 polymorphisms associated with MetS and overcomes the constraints of prior meta-analyses. In addition, the results have been presented based on several subgroups in order to explore the source of heterogeneity and detect any variables that may affect the results of the meta-analysis. Nevertheless, our study, like other genetic meta-analyses, had several limitations that should be taken into account when interpreting the findings of the meta-analysis. Although we analysed the majority of the APOA5 SNPs, it is important to acknowledge that the potential effects of gene-gene interactions were not assessed in the current investigation and should not be disregarded. For example, the interactions between APOA5, BUD13, CETP, and LIPA have been reported to be involved in MetS susceptibility [82]. Aside from the genetic component, environmental and epigenetic factors could play important roles in MetS development [35, 83]. While our subgroup analysis examined the influence of some environmental variables (BMI, age, sex, smoking), the possibility of residual confounding due to unmeasured factors, such as diet or physical activity, cannot be excluded.

Another limitation was the limited number of eligible studies investigating rs651821, rs2075201, and rs126317 polymorphisms, which made it impossible to perform subgroup analysis for these variants.

## Conclusion

We found that polymorphisms located in the promotor and codon regions of the APOA5 gene, including rs662799, rs3135506, rs651821, and rs2075291, were associated with increased MetS prevalence. However, because the sample size was small and there was evidence of significant heterogeneity for some APOA5 gene polymorphisms, these results need to be confirmed by more large-scale and well-designed studies.

### Electronic supplementary material

Below is the link to the electronic supplementary material.

Supplementary Material 1

## Data Availability

The datasets used or analysed during the current study are available from the corresponding author on reasonable request.

## Abbreviations

AHA/NHLBI: American Heart Association/National Heart, Lung and Blood Institute
APOA5: Apolipoprotein A5
APOC3: Apolipoprotein C3
ATP III (NCEP ATP III): National Cholesterol Education Programme Adult Treatment Panel III
BMI: Body Mass Index
CETP: Cholesteryl Ester Transfer Protein
CI: Confidence Interval
DNA: Deoxyribonucleic Acid
IDF: International Diabetes Federation
HDL: High-density Lipoprotein
HDL-C: High-Density Lipoprotein Cholesterol
HWE: Hardy-Weinberg Equilibrium
I^2^: I-squared
JIS: Joint Interim Statement
LDL: Low-density Lipoprotein
LIPA: Lipase A Lysosomal Acid type
LPL: Lipoprotein Lipase
MetS: Metabolic Syndrome
MALDI-TOF: Matrix-Assisted Laser Desorption/Ionization
NOS: Newcastle?Ottawa Scale
OR: Odd Ratio
PCR-RFLP: Polymerase Chain Reaction -Amplification Refractory Mutation System
PCR-SSOP: Polymerase Chain Reaction -Sequence Specific Oligonucleotide Probes
p-value: Probability value
PRISMA: Preferred Reporting Items for Systematic Reviews and Meta-Analyses
SNP: Single-Nucleotide Polymorphism
SNP-IT: SNP Integration Tool
TG: Triglyceride
TNF-α: The Role of Tumor Necrosis Factor Alpha
TRL: Triglyceride-rich Lipoprotein
VLDL: Very Low Density Lipoprotein
