# Supplementary material for: Association between APOA5 polymorphisms and susceptibility to metabolic syndrome: a systematic review and meta-analysis
Source: BMC Genomics. 2024 Jun 12;25:590. doi: 10.1186/s12864-024-10493-x (PMC11167842; doi:10.1186/s12864-024-10493-x)
Supplement: Supplementary file 1 — Supplementary Material 1 [file 12864_2024_10493_MOESM1_ESM.docx]

**Association between APOA5 polymorphisms and susceptibility to metabolic syndrome: a systematic review and meta-analysis**

**Supplementary Table 1.** Search Strategy

| **PubMed** |
| --- |
| “metabolic syndrome” [Mesh terms] OR “metabolic syndrome” [Title/Abstract] OR “Metabolic syndrome x” [Title/Abstract] OR “syndrome X” [Title/Abstract] OR “Dysmetabolic Syndrome” [Title/Abstract] OR “Reaven Syndrome X” [Title/Abstract] OR “Metabolic Cardiovascular Syndrome” [Title/Abstract] OR “Cardiovascular Syndrome” [Title/Abstract] OR “Cardiometabolic Syndrome” [Title/Abstract] OR “cardio metabolic risk factor” [Title/Abstract] OR “cardio-metabolic risk factor” [Title/Abstract] OR “insulin resistance” [Mesh terms] OR “insulin resistance” [Title/Abstract] OR “insulin resistance syndrome” [Title/Abstract]  “Apolipoprotein A-V” [Mesh terms] OR “Apolipoprotein A-V” [Title/Abstract] OR “Apolipoprotein A_V” [Title/Abstract] OR “Apolipoprotein A V” [Title/Abstract] OR “Apolipoprotein AV” [Title/Abstract] OR “Apolipoprotein A5”  [Title/Abstract] OR “APOA-V” [Title/Abstract] OR “APOA_V” [Title/Abstract] OR “APO A-V” [Title/Abstract] OR “APO A_V” [Title/Abstract] OR “APOAV” [Title/Abstract] OR “ApoA V” [Title/Abstract] OR “APO A5” [Title/Abstract] OR “APOA5” [Title/Abstract] OR “rs662799” [Title/Abstract] OR “rs3135506” [Title/Abstract] OR “rs651821” [Title/Abstract] OR “C56G” [Title/Abstract] OR “S19W” [Title/Abstract] OR “-1131T>C” [Title/Abstract] OR “553G>T” [Title/Abstract] OR  “rs2075291” [Title/Abstract] OR “c.553G>T” [Title/Abstract] OR “G553T” [Title/Abstract] OR “Cys185Gly” [Title/Abstract] OR “G185C” [Title/Abstract] OR “G162C” [Title/Abstract]  “Polymorphism, Genetic” [Mesh terms] OR “Polymorphism, Genetic” [Title/Abstract] OR  “polymorphism, single nucleotide” [Mesh terms] OR “polymorphism, single nucleotide” [Title/Abstract] OR Polymorphism [Title/Abstract] OR  “genetic polymorphism” [Title/Abstract] OR  “single nucleotide polymorphism” [Title/Abstract] OR “single nucleotide polymorphisms” [Title/Abstract] OR “SNP” [Title/Abstract] OR “SNPs” [Title/Abstract] OR “SNP” [Title/Abstract] OR “genetic variant” [Title/Abstract] OR “genetic variants” [Title/Abstract] OR “genetic variation” [Title/Abstract] OR Gene [Title/Abstract] OR mutation [Mesh terms] OR mutation [Title/Abstract] OR variant [Title/Abstract] OR variants [Title/Abstract] OR variation [Title/Abstract] OR genotype [Mesh terms] OR genotype [Title/Abstract] |
| **Scopus** |
| TITLE-ABS (“metabolic syndrome” OR “Metabolic syndrome x” OR “syndrome X” OR “Dysmetabolic Syndrome” OR “Reaven Syndrome X” OR “Metabolic Cardiovascular Syndrome” OR “Cardiovascular Syndrome” “Cardiometabolic Syndrome” OR “cardio metabolic risk factor” OR “cardio-metabolic risk factor”  OR “insulin resistance” OR “insulin resistance syndrome”)  TITLE-ABS (“Apolipoprotein A-V” OR “Apolipoprotein A_V” OR “Apolipoprotein A V” OR “Apolipoprotein AV” OR “Apolipoprotein A5” OR “APOA-V” OR “APOA_V” OR “APO A-V” OR “APO A_V” OR “APOAV” OR “ApoA V” OR “APO A5” OR “APOA5” OR “rs662799” OR “rs3135506” OR “rs651821” OR “C56G” OR “S19W” OR “-1131T>C” OR “553G>T” OR “rs2075291” OR “c.553G>T” OR “G553T” OR “Cys185Gly” OR “G185C” OR “G162C”)  TITLE-ABS (“genetic” OR Polymorphism OR “genetic polymorphism” OR “single nucleotide polymorphism” OR “single nucleotide polymorphisms” OR “SNP” OR “SNPs” OR “SNP*” OR “genetic variant” OR “genetic variants” OR “genetic variation” OR Gene OR mutation OR variant OR variants OR variation OR genotype) |
| **Web of Science** |
| TI=(“metabolic syndrome” OR “Metabolic syndrome x” OR “syndrome X” OR “Dysmetabolic Syndrome” OR “Reaven Syndrome X” OR “Metabolic Cardiovascular Syndrome” OR “Cardiovascular Syndrome” “Cardiometabolic Syndrome” OR “cardio metabolic risk factor” OR “insulin resistance” OR “insulin resistance syndrome”) OR AB=(“metabolic syndrome” OR “Metabolic syndrome x” OR “syndrome X” OR “Dysmetabolic Syndrome” OR “Reaven Syndrome X” OR “Metabolic Cardiovascular Syndrome” OR “Cardiovascular Syndrome” “Cardiometabolic Syndrome” OR “cardio metabolic risk factor” OR “insulin resistance” OR “insulin resistance syndrome”)  TI=(“Apolipoprotein A-V” OR “Apolipoprotein A_V” OR “Apolipoprotein A V” OR “Apolipoprotein AV” OR “Apolipoprotein A5” OR “APOA-V” OR “APOA_V” OR “APO A-V” OR “APO A_V” OR “APOAV” OR “ApoA V” OR “APO A5” OR “APOA5” OR “rs662799” OR “rs3135506” OR “rs651821” OR “C56G” OR “S19W” OR “-1131T>C” OR “553G>T” OR “rs2075291” OR “c.553G>T” OR “G553T” OR “Cys185Gly” OR “G185C” OR “p.185Gly>Cys” OR “G162C”) OR AB=(“Apolipoprotein A-V” OR “Apolipoprotein A_V” OR “Apolipoprotein A V” OR “Apolipoprotein AV” OR “Apolipoprotein A5” OR “APOA-V” OR “APOA_V” OR “APO A-V” OR “APO A_V” OR “APOAV” OR “ApoA V” OR “APO A5” OR “APOA5” OR “rs662799” OR “rs3135506” OR “rs651821” OR “C56G” OR “S19W” OR “-1131T>C” OR “553G>T” OR “rs2075291” OR “c.553G>T” OR “G553T” OR “Cys185Gly” OR “G185C” OR “p.185Gly>Cys” OR “G162C”)  TI=(“genetic” OR Polymorphism OR “genetic polymorphism” OR “single nucleotide polymorphism” OR “single nucleotide polymorphisms” OR “SNP” OR “SNPs” OR “SNP*” OR “genetic variant” OR “genetic variants” OR “genetic variation” OR Gene OR mutation OR variant OR variants OR variation OR genotype) OR AB=(“genetic” OR Polymorphism OR “genetic polymorphism” OR “single nucleotide polymorphism” OR “single nucleotide polymorphisms” OR “SNP” OR “SNPs” OR “SNP*” OR “genetic variant” OR “genetic variants” OR “genetic variation” OR Gene OR mutation OR variant OR variants OR variation OR genotype) |
| **Embase** |
| metabolic syndrome/ OR insulin resistance/  (Metabolic syndrome or syndrome X or Dysmetabolic Syndrome or Reaven Syndrome X or Metabolic Cardiovascular Syndrome or Cardiovascular Syndrome or Cardiometabolic Syndrome or cardio metabolic risk factor or insulin resistance syndrome).ti,ab.  Apolipoprotein A-V/  (Apolipoprotein A_V OR Apolipoprotein A V OR Apolipoprotein AV OR Apolipoprotein A5 OR APOA-V OR APOA_V OR APO A-V OR APO A_V OR APOAV OR ApoA V OR APO A5 OR APOA5 OR rs662799 OR rs3135506 OR rs651821 OR C56G OR S19W OR -1131T>C OR 553G>T OR rs2075291 OR G553T OR Cys185Gly OR G185C OR G162C).ti, ab.  Polymorphism, Genetic/ OR polymorphism, single nucleotide/ OR mutation/ OR genotype/  (Polymorphism OR genetic OR single nucleotide polymorphism OR single nucleotide polymorphisms OR SNP OR SNPs OR SNP* OR genetic variant OR genetic variants OR genetic variation OR Gene OR variant OR variants OR variation).ti,ab. |

**Supplementary Table 2. Reasons for exclusion of studies**

| Reasons | References |
| --- | --- |
| Meeting abstract | (1-5) |
| Editorial comment | (6) |
| Review | (7, 8) |
| Not relevant gene | (9, 10) |
| Not relevant outcome | (11-21) |
| Insufficient data | (22-31) |
| Same population | (32-36) |
| Out of age range | (37-39) |

**Supplementary Table 3.** Quality assessment of included studies, using New-Castle Ottawa scale

| **Author** | **Selection Bias** | | | | | | | | **Comparability** | | **Outcome** | | **Score** |
| --- | --- | --- | --- | --- | --- | --- | --- | --- | --- | --- | --- | --- | --- |
|  | Representativeness of sample | | Sample size | | Non-respondents | | Ascertainment exposure | | Comparability | Assessment outcome | | Statistical test |  |
| Ajjemami et al. 2015 | * | -- | | -- | | ** | | * | | ** | | * | high |
| Alipour et al. 2023 | * | * | | -- | | ** | | * | | * | | * | high |
| Dallongeville et al. 2008 | * | * | | -- | | ** | | * | | ** | | * | high |
| de Luis et al. 2021 | * | * | | -- | | ** | | * | | ** | | * | high |
| Fatty et al. 2012 | -- | -- | | -- | | ** | | * | | * | | * | medium |
| Fiaz et al. 2019 | * | * | | -- | | ** | | * | | ** | | * | high |
| Grallert et al. 2007 | * | * | | -- | | ** | | * | | ** | | * | high |
| He et al. 2011 | * | * | | -- | | ** | | -- | | -- | | -- | medium |
| Hechmi et al. 2020 | * | * | | -- | | ** | | * | | ** | | * | high |
| Hiramatsu et al. 2012 | * | * | | -- | | ** | | * | | ** | | * | high |
| Hsu et al. 2008 | * | * | | -- | | ** | | ** | | ** | | * | high |
| Kefi et al. 2017 | * | * | | -- | | ** | | * | | ** | | * | high |
| Kim et al. 2012 | -- | -- | | -- | | ** | | - | | -- | | * | low |
| Kim et al. 2016 | * | * | | -- | | ** | | * | | ** | | * | high |
| Kisfali et al. 2010 | * | * | | -- | | ** | | * | | ** | | * | high |
| Kumurcu-bayrak et al. 2008 | * | * | | -- | | ** | | * | | ** | | * | high |
| Lim et al. 2014 | * | * | | -- | | ** | | * | | -- | | * | medium |
| Lim et al. 2016 | * | * | | -- | | ** | | * | | ** | | -- | high |
| Mattie et al. 2009 | * | * | | -- | | ** | | * | | ** | | * | high |
| Niculescu et al. 2010 | * | -- | | -- | | ** | | * | | * | | * | medium |
| Novotny et al. 2014 | * | * | | -- | | ** | | * | | * | | * | high |
| Ong et al. 2011 | * | * | | -- | | ** | | * | | ** | | * | high |
| Song et al. 2013 | * | * | | -- | | ** | | * | | ** | | * | high |
| Vasilopoulos et al. 2011 | -- | -- | | -- | | ** | | -- | | -- | | -- | low |
| Wu et al. 2016 | * | * | | -- | | ** | | * | | ** | | * | high |
| Xu et al. 2013 | * | * | | -- | | ** | | * | | ** | | * | high |
| Yamada et al. 2007 | * | * | | -- | | ** | | * | | ** | | * | high |
| Yamada et al. 2007 | * | * | | -- | | ** | | * | | -- | | * | medium |
| Yeh et al. 2020 | * | * | | -- | | ** | | * | | ** | | * | high |
| Zafar et al. 2019 | * | -- | | -- | | ** | | * | | ** | | * | high |

**Supplementary Table 4.** Subgroup analyses for the association between APOA polymorphism (rs662799) and metabolic syndrome under the dominant model

|  | **Studies (n)** | **Meta-analysis** | | **Heterogeneity** | | | |
| --- | --- | --- | --- | --- | --- | --- | --- |
|  |  | **OR (95%CI)** | **P effect** | **Q statistic** | **I^2^ (%)** | **P within group** | **P between group** |
| **Continent** |  |  |  |  |  |  | <0.001 |
| Africa | 3 | 3.92 (1.35, 11.39) | 0.01 | 12.09 | 83.5 | 0.002 |  |
| Asia | 3 | 1.56 (1.54, 1.57) | <0.001 | 0.59 | 0.0 | 0.74 |  |
| East Asia | 11 | 1.44 (1.36, 1.53) | <0.001 | 15.30 | 21.6 | 0.22 |  |
| Europe | 6 | 1.21 (0.94, 1.56) | 0.13 | 13.34 | 55.0 | 0.04 |  |
| North America | 1 | 0.87 (0.61, 1.23) | 0.43 | 0.00 | - | - |  |
| **Sex** |  |  |  |  |  |  | <0.001 |
| Male | 5 | 1.37 (1.15, 1.63) | <0.001 | 4.68 | 14.5 | 0.32 |  |
| Female | 8 | 1.41 (1.23, 1.62) | <0.001 | 8.37 | 16.4 | 0.30 |  |
| Both | 24 | 1.42 (1.32, 1.53) | <0.001 | 83.07 | 68.7 | <0.001 |  |
| **MetS definition** |  |  |  |  |  |  | 0.17 |
| IDF | 7 | 1.57 (1.43, 1.73) | <0.001 | 20.80 | 71.2 | 0.002 |  |
| ATP III | 13 | 1.34 (1.14, 1.58) | <0.001 | 33.59 | 61.3 | 0.001 |  |
| IDF-modified | 1 | 1.34 (1.10, 1.63) | 0.004 | 2.00 | 50.0 | 0.16 |  |
| ATP-modified | 2 | 1.49 (1.27, 1.73) | <0.001 | 0.82 | 0.0 | 0.37 |  |
| Harmonized definition | 1 | 1.28 (1.08, 1.51) | 0.004 | 0.10 | 0 | 0.75 |  |
| **HWE** |  |  |  |  |  |  | 0.24 |
| Yes | 20 | 1.39 (1.31, 1.49) | <0.001 | 60.21 | 63.5 | <0.001 |  |
| No | 1 | 1.50 (1.01, 2.23) | 0.045 | - | - | - |  |
| NM | 3 | 3.75 (1.16, 12.10) | 0.027 | 14.72 | 86.4 | 0.001 |  |
| **Adjustments** |  |  |  |  |  |  |  |
| Age |  |  |  |  |  |  | 0.08 |
| Yes | 9 | 1.52 (1.39, 1.66) | <0.001 | 28.29 | 71.7 | <0.001 |  |
| No | 15 | 1.34 (1.19, 1.50) | <0.001 | 39.18 | 56.6 | 0.002 |  |
| Sex |  |  |  |  |  |  | 0.08 |
| Yes | 9 | 1.52 (1.39. 1.66) | <0.001 | 28.29 | 71.7 | <0.001 |  |
| No | 15 | 1.34 (1.19, 1.50) | <0.001 | 39.18 | 56.6 | 0.002 |  |
| BMI |  |  |  |  |  |  | <0.001 |
| Yes | 1 | 7.82 (3.79, 16.14) | <0.001 | 0.0 | - | - |  |
| No | 23 | 1.40 (1.32, 1.50) | <0.001 | 63.96 | 60.9 | <0.001 |  |
| Smoking |  |  |  |  |  |  | 0.19 |
| Yes | 2 | 1.60 (1.34, 1.91) | <0.001 | 0.23 | 0 | 0.63 |  |
| No | 22 | 1.41 (1.30, 1.52) | <0.001 | 82.73 | 71.0 | <0.001 |  |
| **Quality Score** |  |  |  |  |  |  | 0.57 |
| Good | 19 | 1.40 (1.30, 1.51) | <0.001 | 73.00 | 71.2 | <0.001 |  |
| Moderate | 3 | 1.59 (0.81, 3.12) | 0.18 | 7.24 | 72.4 | 0.03 |  |
| Poor | 2 | 2.07 (0.97, 4.43) | 0.06 | 2.28 | 56.2 | 0.13 |  |
| **Sample size** |  |  |  |  |  |  | 0.015 |
| <200 | 2 | 3.95 (1.90, 8.18) | <0.001 | 0.23 | 0.0 | 0.63 |  |
| 200< >500 | 4 | 1.91 (0.92, 3.99) | 0.08 | 22.22 | 86.5 | <0.001 |  |
| >500 | 18 | 1.42 (1.31, 1.49) | <0.001 | 53.87 | 62.9 | <0.001 |  |

OR, Odd Ratio; P, P-value; I^2^, I-squared; MetS, Metabolic Syndrome; HWE, Hardy–Weinberg Equilibrium; NM, Not mentioned; ATP III (NCEP ATP III), National Cholesterol Education Programme Adult Treatment Panel III; IDF, International Diabetes Federation; BMI, Body Mass Index.

**Supplementary Table 5.** Subgroup analyses for the association between APOA polymorphism (rs3135506) and metabolic syndrome under the dominant model

|  | Studies (n) | Meta-analysis | | Heterogeneity | | | |
| --- | --- | --- | --- | --- | --- | --- | --- |
|  |  | OR (95%CI) | P effect | Q statistic | I^2^ (%) | P within group | P between group |
| **Continent** |  |  |  |  |  |  | 0.20 |
| Africa | 2 | 1.59 (1.09, 2.33) | 0.02 | 0.90 | 0.0 | 0.34 |  |
| Europe | 5 | 1.40 (1.21, 1.62) | <0.001 | 1.11 | 0.0 | 0.95 |  |
| North America | 1 | 1.02 (0.71, 1.47) | 0.92 | 0 | - | - |  |
| **Sex** |  |  |  |  |  |  | 0.47 |
| Female |  | 1.58 (0.99, 2.53) | 0.06 | 0 | - | - |  |
| Both |  | 1.34 (1.17, 1.52) | <0.001 | 5.95 | 0.65 | 0 |  |
| **MetS definition** |  |  |  |  |  |  | 0.41 |
| IDF | 2 | 1.59 (1.09, 2.33) | 0.02 | 0.90 | 0.0 | 0.34 |  |
| ATP III | 6 | 1.34 (1.17, 1.54) | <0.001 | 3.59 | 0.0 | 0.73 |  |
| **HWE** |  |  |  |  |  |  | 0.21 |
| Yes | 7 | 1.35 (1.18, 1.53) | <0.001 | 3.62 | 0.0 | 0.82 |  |
| NM | 1 | 2.07 (1.07, 4.02) | 0.03 | 0 | - | - |  |
| **Adjustments** |  |  |  |  |  |  |  |
| Age |  |  |  |  |  |  | 0.94 |
| Yes | 3 | 1.38 (1.12, 1.68) | 0.002 | 1.68 | 0.0 | 0.43 |  |
| No | 5 | 1.36 (1.16, 1.60) | <0.001 | 3.49 | 0.0 | 0.62 |  |
| Sex |  |  |  |  |  |  | 0.94 |
| Yes | 3 | 1.38 (1.12, 1.68) | 0.002 | 1.68 | 0.0 | 0.43 |  |
| No | 5 | 1.36 (1.16, 1.60) | <0.001 | 3.49 | 0.0 | 0.62 |  |
| BMI |  |  |  |  |  |  | 0.17 |
| Yes | 2 | 1.59 (1.09, 2.33) | 0.02 | 0.90 | 0.0 | 0.34 |  |
| No | 6 | 1.34 (1.17, 1.54) | <0.001 | 3.59 | 0.0 | 0.73 |  |
| **Quality Score** |  |  |  |  |  |  | 0.47 |
| Good | 7 | 1.36 (1.19, 1.54) | <0.001 | 4.66 | 0.0 | 0.70 |  |
| Moderate | 1 | 1.78 (0.86, 3.68) | 0.12 | 0.0 | - | - |  |
| **Sample size** |  |  |  |  |  |  | 0.15 |
| 200< >500 | 2 | 1.93 (1.18, 3.15) | 0.008 | 0.09 | 0.0 | 0.76 |  |
| >500 | 6 | 1.33 (1.17, 1.52) | <0.001 | 3.04 | 0.0 | 0.80 |  |

OR, Odd Ratio; P, P-value; I^2^, I-squared; MetS, Metabolic Syndrome; HWE, Hardy–Weinberg Equilibrium; ATP III (NCEP ATP III), National Cholesterol Education Programme Adult Treatment Panel III; IDF, International Diabetes Federation; BMI, Body Mass Index.

**References**:

1. Warodomwichit D, Ordovas JM, Sritara P, Sura T, Jongjirasiri S, Laothamatas J, et al. APOA5-1131T > C POLYMORPHISM, LIPIDS, METABOLIC SYNDROME AND CARDIOVASCULAR DISEASE IN THAIS. Annals of Nutrition and Metabolism. 2009;55:447-.

2. Jiang CQ, Liu B, Lin JM, Yue XJ, Ong KL, Tam S, et al. A single nucleotide polymorphism in the APOA5 gene is associated with the metabolic syndrome. Journal of Diabetes. 2009;1(SUPPL. 1):A4.

3. Bielicki P, Barnas M, Brzoska K, Jonczak L, Plywaczewski R, Kumor M, et al. Genetic predisposition to arterial hypertension in patients with OSA. Eur Respiratory Soc; 2013.

4. Warodomwichit D, Sritara P, Sura T, Jongjirasiri S, Laothamatas J, Yamwong S, et al. APOA5 -1131T>C polymorphism modulates plasma triglyceride levels and contributes to risk of metabolic syndrome and coronary artery disease in Thais. European Heart Journal. 2009;30(SUPPL. 1):772.

5. Niculescu L, Vladica M, Sima A. APOA5 and APOC3 genotypes in patients with dyslipidemia and insulin resistance. Atherosclerosis Supplements. 2009;10(2).

6. Seo MH, Lee WY. APOA5 Polymorphism Is Associated with Metabolic Syndrome in Korean Postmenopausal Women. Endocrinology and Metabolism. 2012;27(4):274-5.

7. Liu CF, Yang QF, Chen XL, Liu CY. Apolipoprotein A5 gene polymorphism and risk for metabolic syndrome: A meta-analysis. Genetic Testing and Molecular Biomarkers. 2012;16(10):1241-5.

8. Shojaei S, Daneshpour M, Halalkhor S, Azizi F, Hedayati M. Genetic association between metabolic syndrome and apolipoproteins. Iranian Journal of Endocrinology and Metabolism. 2011;13(2):209.

9. Šedová L, Liška F, Křenová D, Kazdová L, Tremblay J, Krupková M, et al. CD36-deficient congenic strains show improved glucose tolerance and distinct shifts in metabolic and transcriptomic profiles. Heredity. 2012;109(1):63-70.

10. Ghazizadeh H, Avan A, Fazilati M, Azimi-Nezhad M, Tayefi M, Ghasemi F, et al. Association of rs6921438 A<G with serum vascular endothelial growth factor concentrations in patients with metabolic syndrome. Gene. 2018;667:70-5.

11. Jeong SW, Chung M, Park SJ, Cho SB, Hong KW. Genome-wide association study of metabolic syndrome in koreans. Genomics Inform. 2014;12(4):187-94.

12. Chien KL, Hsu HC, Chen YC, Su TC, Lee YT, Chen MF. Association between sequence variant of c.553 G > T in the apolipoprotein A5 gene and metabolic syndrome, insulin resistance, and carotid atherosclerosis. Translational Research. 2009;154(3):133-41.

13. Shojaei S, Halalkhor S, Hajian K, Jalali F. Association of two common polymorphisms of APOA5 with lipid profile in the north of Iran. Obesity Reviews. 2010;11(SUPPL. 1):149.

14. Lin YC, Nunez V, Johns R, Shiao SP. APOA5 Gene Polymorphisms and Cardiovascular Diseases: Metaprediction in Global Populations. Nursing research. 2017;66(2):164-74.

15. Charlton-Menys V, Durrington PN. Apolipoprotein A5 and hypertriglyceridemia. Clinical Chemistry. 2005;51(2):295-7.

16. Cabré A, Lázaro I, Girona J, Manzanares JM, Marimón F, Plana N, et al. The APOA5-1131 T>C variant enhances the association between RBP4 and hypertriglyceridemia in diabetes. Nutr Metab Cardiovasc Dis. 2010;20(4):243-8.

17. Halalkhor S, Jalali F, Tilaki KH, Shojaei S. Association of two common polymorphisms of apolipoprotein A5 gene with metabolic syndrome indicators in a North Iranian population, a cross-sectional study. J Diabetes Metab Disord. 2014;13:1-7.

18. Tabb KL, Hellwege JN, Palmer ND, Dimitrov L, Sajuthi S, Taylor KD, et al. Analysis of Whole Exome Sequencing with Cardiometabolic Traits Using Family-Based Linkage and Association in the IRAS Family Study. Ann Hum Genet. 2017;81(2):49-58.

19. Hadarits F, Kisfali P, Mohas M, Maasz A, Duga B, Janicsek I, et al. Common functional variants of APOA5 and GCKR accumulate gradually in association with triglyceride increase in metabolic syndrome patients. Molecular Biology Reports. 2012;39(2):1949-55.

20. Carty CL, Bhattacharjee S, Haessler J, Cheng I, Hindorff LA, Aroda V, et al. Analysis of Metabolic Syndrome Components in > 15 000 African Americans Identifies Pleiotropic Variants Results From the Population Architecture Using Genomics and Epidemiology Study. Circulation-Cardiovascular Genetics. 2014;7(4):505-U303.

21. Zhou G, Liu B. Single nucleotide polymorphisms of metabolic syndrome-related genes in primary open angle glaucoma. International Journal of Ophthalmology. 2010;10(1):23-9.

22. Oguri M, Shibata Y, Kamiya H, Hiramatsu M, Fujimaki T, Kato K, et al. Effects of combined genotypes for polymorphisms of the apolipoprotein A-V gene and the butyrophilin, subfamily 2, member A1 gene on metabolic syndrome in east Asian populations. European Heart Journal. 2012;33(SUPPL. 1):879.

23. Song KH, Cha S, Yu S-G, Yu H, Oh SA, Kang N-S. Association of Apolipoprotein A5 Gene 1131T C Polymorphism with the Risk of Metabolic Syndrome in Korean Subjects. bmri. 2013;2013.

24. Novotny D, Karasek D, Vaverkova H, Bartkova M, Kubickova V. Paraoxonase 2 Cys311Ser polymorphism and its association with the systolic blood pressure values in asymptomatic dyslipidemic individuals: A pilot study. LaboratoriumsMedizin. 2015;39(4):249-57.

25. Cha S, Yu H, Park AY, Song KH. Effects of apolipoprotein A5 haplotypes on the ratio of triglyceride to high-density lipoprotein cholesterol and the risk for metabolic syndrome in Koreans. Lipids in Health and Disease. 2014;13(1):45.

26. Fallah MS, Sedaghatikhayat B, Guity K, Akbari F, Azizi F, Daneshpour MS. The relation between metabolic syndrome risk factors and genetic variation in apolipoprotein V in relation with serum triglyceride and HDL-C level. Archives of Iranian Medicine. 2016;19(1):32-6.

27. Singmann P, Baumert J, Herder C, Meisinger C, Holzapfel C, Klopp N, et al. Gene-Gene Interaction between APOA5 and USF1: Two Candidate Genes for the Metabolic Syndrome. Obesity Facts. 2009;2(4):235-42.

28. Nakatochi M, Ushida Y, Yasuda Y, Yoshida Y, Kawai S, Kato R, et al. Identification of an interaction between VWF rs7965413 and platelet count as a novel risk marker for metabolic syndrome: An extensive search of candidate polymorphisms in a case-control study. PLoS ONE. 2015;10(2):e0117591.

29. Kvaløy K, Holmen J, Hveem K, Holmen TL. Genetic Effects on Longitudinal Changes from Healthy to Adverse Weight and Metabolic Status – The HUNT Study. PLoS One. 2015;10(10):e0139632.

30. Hubacek JA, Lanska V, Adamkova V. Common apoa5 haplotype is associated with metabolic syndrome in Czech females but not in males. Journal of Diabetes. 2013;5(SUPPL. 1):142.

31. Zhu Y, Zhang D, Zhou D, Li Z, Li Z, Fang L, et al. Susceptibility loci for metabolic syndrome and metabolic components identified in Han Chinese: a multi‐stage genome‐wide association study. Journal of cellular and molecular medicine. 2017;21(6):1106-16.

32. Maász A, Kisfali P, Horvatovich K, Mohás M, Markó L, Csöngei V, et al. Apolipoprotein A5 T-1131C variant confers risk for metabolic syndrome. Pathol Oncol Res. 2007;13(3):243-7.

33. Jiang CQ, Liu B, Cheung BM, Lam TH, Lin JM, Li Jin Y, et al. A single nucleotide polymorphism in APOA5 determines triglyceride levels in Hong Kong and Guangzhou Chinese. Eur J Hum Genet. 2010;18(11):1255-60.

34. Lin E, Kuo PH, Liu YL, Yang AC, Tsai SJ. Detection of susceptibility loci on APOA5 and COLEC12 associated with metabolic syndrome using a genome-wide association study in a Taiwanese population. Oncotarget. 2017;8(55):93349-59.

35. Lin E, Kuo PH, Liu YL, Yang AC, Kao CF, Tsai SJ. Association and interaction of APOA5, BUD13, CETP, LIPA and health-related behavior with metabolic syndrome in a Taiwanese population. Scientific reports. 2016;6:36830.

36. Kisfali P, Mohas M, Maasz A, Hadarits F, Marko L, Horvatovich K, et al. Apolipoprotein A5 IVS3+476A allelic variant associates with increased trigliceride levels and confers risk for development of metabolic syndrome in Hungarians. Circulation Journal. 2008;72(1):40-3.

37. Salehi S, Emadi-Baygi M, Rezaei M, Kelishadi R, Nikpour P. Identification of a New Single-nucleotide Polymorphism within the Apolipoprotein A5 Gene, Which is Associated with Metabolic Syndrome. Adv Biomed Res. 2017;6:24.

38. Fatemi SG, Emadi-Baygi M, Nikpour P, Kelishadi R, Hashemipour M. Absence of association between -1131T>C polymorphism in the apolipoprotein APOA5 gene and pediatric metabolic syndrome. Iranian Journal of Pediatrics. 2014;24(3):319-22.

39. Zaki M, Amr K. Apolipoprotein A5 T-1131C variant and risk for metabolic syndrome in obese adolescents. Gene. 2014;534(1):44-7.
